# Epidemic Hand, Foot and Mouth Disease Caused by Human Enterovirus 71, Singapore

**DOI:** 10.3201/eid1301.020112

**Published:** 2003-01

**Authors:** Kwai Peng Chan, Kee Tai Goh, Chia Yin Chong, Eng Swee Teo, Gilbert Lau, Ai Ee Ling

**Affiliations:** * Singapore General Hospital, Singapore, Republic of Singapore; † National Environment Agency, Singapore, Republic of Singapore,; ‡ Kandang Kerbau Women’s and Children’s Hospital, Singapore, Republic of Singapore; § Centre for Forensic Medicine, Health Sciences Authority, Singapore, Republic of Singapore

**Keywords:** hand, foot and mouth disease, human enterovirus infection, HEV71, encephalitis, myocarditis, pneumonitis, research

## Abstract

Singapore experienced a large epidemic of hand, foot and mouth disease (HFMD) in 2000. After reviewing HFMD notifications from doctors and child-care centers, we found that the incidence of HFMD rose in September and declined at the end of October. During this period, 3,790 cases were reported. We performed enteroviral cultures on 311 and 157 specimens from 175 HFMD patients and 107 non-HFMD patients, respectively; human enterovirus 71 (HEV71) was the most frequently isolated virus from both groups. Most of the HFMD patients were <4 years of age. Three HFMD and two non-HFMD patients died. Specimens from two HFMD and both non-HFMD patients were culture positive for HEV71; a third patient was possibly associated with the virus. Autopsies performed on all three HFMD and one of the non-HFMD case-patients showed encephalitis, interstitial pneumonitis, and myocarditis. A preparedness plan for severe HFMD outbreaks provided for the prompt, coordinated actions needed to control the epidemic.

Hand, foot and mouth disease (HFMD) is typically a benign and common self-limiting childhood disease, characterized by rapidly ulcerating vesicles in the mouth and lesions, usually vesicular, on the hands and feet (*1*). Lesions also frequently occur on the buttocks, but other parts of the body are usually not affected (*2*). HFMD is caused by a few serotypes of enteroviruses, most frequently coxsackie virus A16 (CAV16) and human enterovirus 71 (HEV71). Other viruses associated with the syndrome are coxsackie virus A (CAV) 4, 5, 9, and 10 and coxsackie virus B (CBV) 2 and 5 (*1*). The first recognized HFMD outbreak in Singapore occurred in 1970; the etiologic agent was unknown (*3*). Two other outbreaks were reported in 1972 and 1981 and involved 104 and 742 persons, respectively; in both outbreaks, CAV16 was implicated as the cause (*4*,*5*).

After epidemics of HFMD in Sarawak, East Malaysia, and the Malaysian Peninsula in 1997 (*6*–*8*) and Taiwan in 1998 (*9*,*10*), which were associated with complications of encephalitis, myocarditis, and death, a system of surveillance for the disease, based on notifications from child-care centers, was implemented in Singapore in April 1998. Reporting the disease was made legally mandatory on October 1, 2000. Concurrent with the intensified surveillance, an interministry and interhospital HFMD Task Force, composed of representatives from the Ministries of Health, Environment, Education, and Community Development and Sports, as well as virologists and pediatricians, was created in 1998 to formulate a preparedness response plan to monitor and manage severe HFMD outbreaks in Singapore.

At the end of 2000, Singapore experienced its largest known outbreak of HFMD. After media reports in September of HFMD-related deaths in Singaporean children, many patient samples were sent for virologic investigation to the Virology Laboratory of the Department of Pathology, Singapore General Hospital. Because the Virology Laboratory receives all requests for virus culture or enterovirus typing from the entire country, it was the repository of information on virtually all laboratory investigations during the HFMD epidemic. We describe the epidemiologic, virologic, and pathologic features of this epidemic.

## Methods

In this study, we used a case definition for HFMD of fever, accompanied by oral ulcers and a rash, maculopapular or vesicular, on the hands and feet, with or without buttock involvement. We reviewed records of HFMD notifications to the Ministry of the Environment for the incidence and trend of the disease. All children with suspected HFMD reported by preschool centers were examined, and the cases were certified by family physicians. Cases reported by parents or school principals and teachers were excluded unless a medical certificate from a physician verified them. At the same time, Ministry of the Environment nurses conducted active case detection in both preschools and primary schools. All case-patients were identified by a unique national registration identification number, and duplicate reports were eliminated by the computer.

Data obtained from samples received by the Virology Laboratory at Singapore General Hospital for enterovirus isolation during the epidemic were also analyzed. In addition to stool samples, samples included swabs of vesicles, mouth, throat, rectum, and ulcers, and samples from the brain, heart, lung, tonsil, lymph node, spleen, and intestine of those with fatal disease. The samples were added into HeLa, HEp-2, human embryonic lung fibroblasts, and human rhabdomyosarcoma cells. The cultures were incubated at 36°C and examined daily for cytopathic effects for 21 to 28 days.

Enteroviruses cultured from the samples was typed by micro-neutralization tests (*11*) by using Lim Benyesh-Melnick A-H equine antiserum pools (World Health Organization, Statens Serum Institut, Copenhagen, Denmark), equine antiserum pools (Rijksinstituut voor Volksgesondheid en Milieuhygiene, Bilthoven, the Netherlands), rabbit 385JS HEV71-specific polyclonal antiserum (Victorian Infectious Diseases Reference Laboratory, Melbourne, Australia), and rabbit or monkey antisera specific for CAV serotypes (National Institutes of Health, Bethesda, MD). Nonenteroviruses that produced cytopathic effects characteristic of *Cytomegalovirus* (CMV) or herpes simplex virus were identified by immunofluorescence assay as described (*12*), by using mouse monoclonal antibodies to CMV (Bartels CMV DFA kit, Trinity Biotech plc, Wicklow, Ireland) and herpes simplex virus (MicroTrak HSV1/HSV2 culture identification/typing test, Trinity Biotech plc). When the presence of rhinovirus was suggested from the cytopathic effects, the virus was identified by the acid lability test (*13*). Autopsies were performed on four patients who died, and tissue samples were subjected to virus cultures.

## Results

### Cases

**Figure 1:**
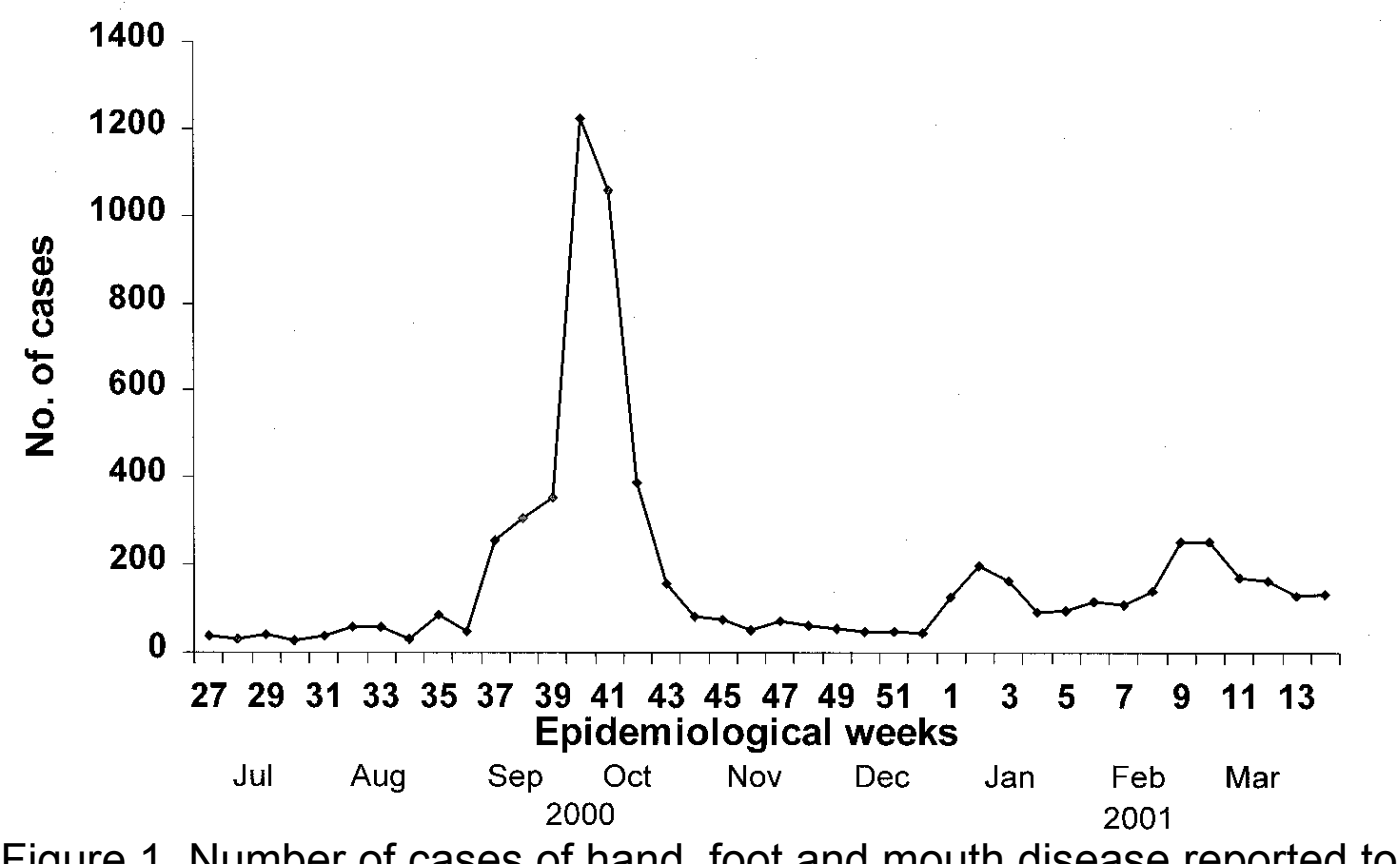
Number of cases of hand, foot and mouth disease reported to the Singapore Ministry of Environment as surveillance for the disease, July 2000–March 2001. Each epidemiologic week begins on Sunday. Mandatory reporting of the disease began on October 1, 2000.

The number of notifications of HFMD cases to the Ministry of the Environment increased in early September 2000 (Figure 1). The incidence peaked at 308 cases per day on October 10 and decreased to 10 cases per day by October 28. Hospital and general practice physicians and preschool-center operators reported a total of 3,790 cases during these 2 months.

During the epidemic, 311 samples from 175 clinically diagnosed HFMD case-patients were submitted for virus culture. A total of 138 (78.8%) of these patients were <4 years of age, with 12 (6.9%) >10 years of age, the oldest being 71 years old (Table 1). The male-to-female ratio was 1.7:1.

**Table 1:** Age distribution of clinical and virus-positive hand, foot and mouth disease patients

Age (yrs)	No. clinical cases (%)	No. virus-positive cases (%)
<1	16 (9.1)	11 (10.6)
1	44 (25.2)	32 (30.8)
2	41 (23.4)	24 (23.1)
3	21 (12.0)	14 (13.5)
4	16 (9.1)	8 (7.7)
5	8 (4.6)	6 (5.8)
6	6 (3.4)	4 (3.9)
7	4 (2.3)	1 (0.9)
8	3 (1.7)	2 (1.9)
9	1 (0.6)	0 (0.0)
10	3 (1.7)	1 (0.9)
>10	12 (6.9)	1 (0.9)
Total	175 (100.0)	104 (100.0)

At least one virus was isolated from 147 (47.3%) of the samples collected from 104 (59.4%) HFMD patients, including 2 of 3 who died. Almost all (91.5%) of these patients were <5 years of age with the peak incidence at 1 year (Table 1). A 21-year-old woman was the only patient >10 years of age to yield a virus, identified as HEV71, from vesicles on her hands and feet.

HEV71 was the most commonly isolated virus, detected in 76 (73.1%) of 104 case-patients (Table 2). Three of these patients had a second virus isolated concurrently: echovirus (EV) 25, *Rhinovirus*, and CMV. Other enteroviruses were isolated in 24 (23.1%) of samples from case-patients. Five cases of CAV16 were identified, as well as four cases each of CAV6, CAV24, and EV18; three cases of CAV10; and one case each of CAV4, CBV3, CBV4, and CBV5. Four patients (3.8%) tested positive for nonenteroviruses; CMV was isolated from their mouth and from throat swabs.

**Table 2:** Viruses isolated from HFMD cases during the epidemic^a^

Virus	No. HFMD patients (%)	No. non-HFMD patients (%)
HEV71 HEV71 only HEV71 + EV25 HEV71 + *Rhinovirus* HEV71 + CMV	76 (73.1) 73 1 1 1	5 (29.4) 5 0 0 0
CAV4	1 (1.0)	0
CAV6	4 (3.8)	0
CAV10	3 (2.9)	1 (5.9)
CAV16	5 (4.8)	0
CAV24	4 (3.8)	3 (17.6)
CBV3	1 (1.0)	1 (5.9)
CBV4	1 (1.0)	2 (11.8)
CBV5	1 (1.0)	3 (17.6)
EV18	4 (3.8)	0
CMV	4 (3.8)	0
Herpes simplex virus 1	0	2 (11.8)
Total	104 (100.0)	17 (100.0)

^a^HFMD, hand, foot and mouth disease; HEV, human enterovirus; EV, echovirus; CMV, *Cytomegalovirus*; CAV, coxsackie virus A; CBV, coxsackie virus B.

**Figure 2:**
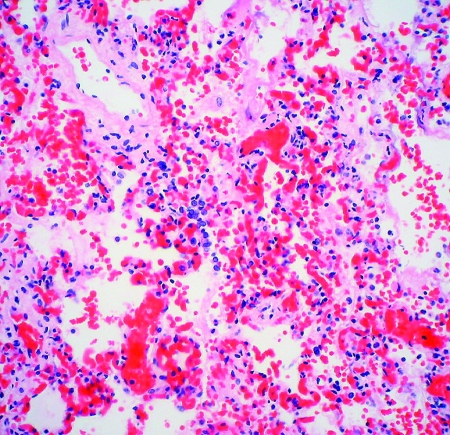
Interstitial pneumonitis in the 14-month-old girl who died of human enterovirus 71 disease. Photomicrograph shows alveolar wall congestion, intra-alveolar hemorrhage, and interstitial lymphocytic infiltrate. (Hematoxylin and eosin stain, original magnification x 200).

**Figure 3:**
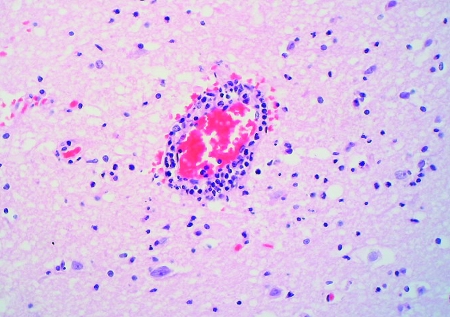
Perivascular cuffing in the brain. (Hematoxylin and eosin stain, original magnification x 200).

**Figure 4:**
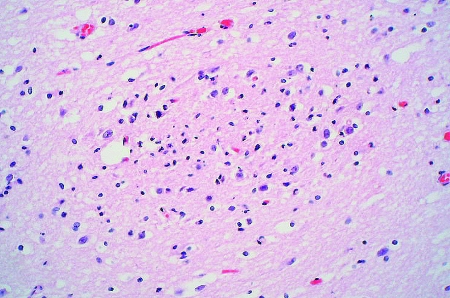
Section of brain showing a focus of necrosis. (Hematoxylin and eosin stain, original magnification x 200).

**Figure 5:**
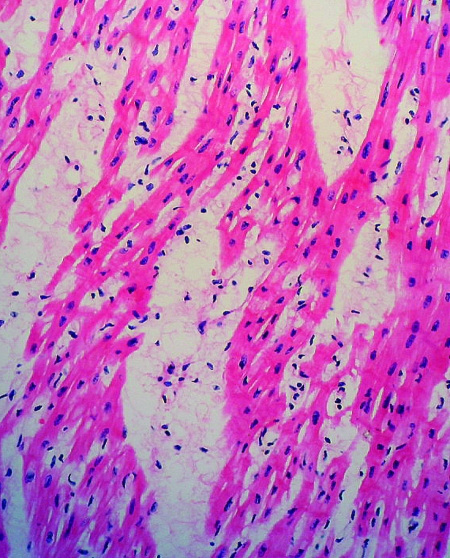
Tissue section of heart showing lymphocytic infiltrate, interstitial edema, and myocardial necrosis. (Hematoxylin and eosin stain, original magnification x200).

The two patients with fatal HFMD, from whom HEV71 was isolated, were siblings, a 14-month-old girl and her 2-year-old brother. The girl was admitted to the hospital with fever, rashes on the hands and feet, and oral ulcers of 3 days’ duration. Progressive hemodynamic instability, oliguria, metabolic acidosis, and hyperkalemia developed; despite intensive care and resuscitative efforts, she died on day 2 after admission. At autopsy, her lungs showed acute pulmonary edema, acute intraalveolar hemorrhage and diffuse alveolar damage associated with interstitial lymphocytic infiltrates, extensive hyaline membrane formation, patchy atelectasis, and focal pneumocyte desquamation and hypertrophy (Figure 2). Samples from her brain tissue showed lymphocytic leptomeningitis with widespread perivascular cuffing by lymphocytes and plasma cells within the cortex and white matter (Figure 3). The pons, in particular, showed evidence of encephalitis, associated with localized perivascular hemorrhage, focal neuronal necrosis, and microglial reaction (Figure 4). Features of myocarditis were observed; the myocardium showed occasional interstitial infiltrates of lymphocytes and plasma cells associated with focal myonecrosis (Figure 5). HEV71 was isolated from samples taken from the brain, tonsils, intestines, stools, and throat and from swabs of the mouth and rectum. Viral cultures of the lung, heart, and spleen were negative.

This patient had two older brothers. The 2-year-old brother showed an almost identical clinical course. After 3 days of fever, rash on the hands and feet, and oral ulcers, he too deteriorated under intensive care and died about 2 hours after his sister, within 24 hours of hospitalization. Autopsy findings were similar, showing evidence of acute interstitial pneumonitis, pulmonary edema, encephalitis (including focal neuronal necrosis of the pons), and myocarditis. HEV71 was isolated from postmortem specimens of the tonsils and intestines. Viral cultures of the brain, heart, lungs, spleen, and lymph nodes showed negative results.

The 5-year-old brother of these patients was also admitted to the hospital at the same time with rashes and mouth ulcers, which in his case did not progress to severe disease. However, because of the rapid deaths of his siblings, intravenous immunoglobulin was administered prophylactically 2 days after hospital admission. After two doses, vomiting and a headache developed. A computed tomographic scan of his head showed normal results, and after due assessment, the headache and vomiting were attributed to the intravenous immunoglobulin, which was then stopped. The symptoms ceased and the boy recovered well. HEV71 was cultured from his throat swab and stool.

Another 2-year-old boy with 5 days of fever, cough, and rash on the hands and feet was diagnosed with HFMD and died on the same day he was admitted to the hospital. Postmortem examination showed pulmonary edema, interstitial pneumonitis, leptomeningeal infiltrates of lymphocytes and plasma cells, and occasional foci of perivascular lymphoid cuffing within the cerebral cortex. Although the heart contained patchy epicardial lymphoid infiltrates, no evidence of myonecrosis was found. Virus was not isolated from the brain, heart, lungs, intestine, and tracheal swab specimen.

One other complicated HFMD case was seen. Aseptic meningitis manifested by headache and terminal neck stiffness developed in an 8-year-old girl with characteristic symptoms of HFMD. HEV71 was isolated from her oral ulcers, but her cerebrospinal fluid (CSF) was negative for viruses and bacteria. After she was treated for her symptoms, the patient was discharged from the hospital after 4 days. No patient in this epidemic showed acute flaccid paralysis.

Over the same period, 107 other patients who did not have HFMD were investigated for enteroviral infection. HEV71 was isolated from 5 of 17 culture-positive patients (Table 2). Of the five HEV71 patients, three had a nonspecific febrile illness without rash. The other two patients died. One was a 1-year-old boy with a 3-day history of fever, vomiting, diarrhea, and increasing restlessness; he died the day after being admitted to the hospital. Tests for bacteria, *Dengue virus*, *Rotavirus*, and *Plasmodium* spp. were negative; however, HEV71 was isolated from his throat and rectal swabs. Cardiac enzyme levels were raised, and a clinical diagnosis of myocarditis was made. No autopsy was performed. The other patient who died was a 19-year-old man who had a fever and headache for 3 days, associated with slurred speech and an episode of generalized tonic-clonic seizure. He died about 16 hours after hospital admission in spite of maximum resuscitative efforts. Postmortem investigation found meningoencephalitis involving the cerebral cortex and pons. The latter also showed focal liquefactive necrosis. His lungs showed marked intraalveolar hemorrhage and edema, and enlarged pneumocytes with intense nuclear smudging consistent with acute interstitial pneumonitis. The myocardium did not show notable inflammation or necrosis. The culture of brain tissue showed HEV71, but cultures of his heart, lungs, and intestines were negative for viruses.

Non-HEV71 enteroviruses were cultured from patients with hemorrhagic conjunctivitis (CAV24), aseptic meningitis (CBV4, CBV5), neonatal pyrexia (CBV3, CBV4), gastroenteritis (CAV24), sudden infant death (CAV24), and pharyngitis (CAV10) (Table 2). Two patients in whom herpangina was diagnosed had herpes simplex virus type 1 cultured from their oral swabs.

### Specimens

The majority of specimens received from HFMD case-patients included those from stool, vesicles, and mouth and throat swabs (Table 3), for which the HEV71 culture-positivity rate was 44.3%, 43.6%, 25.0%, and 32.0% respectively. For the non-HFMD patients, stool and CSF specimens were most frequently submitted; 5.4% of the stool specimens and none of the CSF specimens were HEV71 positive. Non-HEV71 viruses, however, were cultured from five CSF specimens; one yielded CBV3, two yielded CBV4, and two yielded CBV5.

**Table 3:** Virus yield by specimen type^a^

Specimen type	No. specimens					
	HFMD patients			Non-HFMD patients		
	Culture +	HEV 71 +	No. tested	Culture +	HEV 71 +	No. tested
Stool	58	39^b^	88	11	4	74
Rectal swab	6	5	8	1	1	3
Vesicle	31	27	62	0	0	3
Oral swab	23	15	60	2	0	6
Throat swab	19	16^c^	50	2	1	9
Nasal aspirate	3	3^d^	6	1	1	1
Saliva	1	1^d^	3	0	0	0
Tonsil	2	2	2	0	0	0
Intestine and contents	2	2	5	1	0	6
Brain	1	1	3	1	1	7
Cerebrospinal fluid	0	0	2	5	0	32
Ulcer	1	1	2	0	0	0
Conjunctiva	0	0	1	1	0	1
Blood	0	0	2	0	0	0
Heart	0	0	4	0	0	12
Tracheal swab	0	0	4	0	0	1
Lymph node	0	0	1	0	0	0
Spleen	0	0	2	0	0	0
Lung	0	0	4	0	0	1
Nasal swab	0	0	2	0	0	0
BAL	0	0	0	0	0	1
Total	147	112	311	25	8	157

^a^HFMD, hand, foot and mouth disease; HEV, human enterovirus; +, positive; EV, echovirus; CMV, *Cytomegalovirus*; BAL, broncho-alveolar lavage. ^b^Dual isolation of HEV71 and EV25 from one specimen. ^c^Dual isolation of HEV71 and *Rhinovirus* from one specimen. ^d^Dual isolation of HEV71 and CMV from one specimen. Both double-virus positive specimens were received from the same patient.

## Discussion

The HFMD epidemic of 2000 is remarkable for differing from previous outbreaks in Singapore in three ways: the size of the epidemic, the causative virus, and the deaths associated with the epidemic. In these aspects, this outbreak is similar to those that occurred in Malaysia and Taiwan in recent years (*6*,*7*,*9*,*10*).

Cases in previous Singaporean outbreaks had numbered in the hundreds (*3*–*5*), contrasting with the approximately 4,000 cases reported in September and October 2000. The large number could in part have been the result of the HFMD surveillance initiated in 1998. In addition, physicians, parents, and child-care givers had a heightened awareness of the disease as a result of media publicity over the local HFMD-related deaths in September. Parents and caregivers sought medical attention for many children, including for those with mild illness. Another contributing factor was the compulsory reporting of the disease beginning on October 1, 2000.

HEV71 was isolated from 73.1% of the virus-positive HFMD patients and was the most probable cause of the epidemic, unlike the earlier documented outbreaks in Singapore (*4*,*5*), which were attributed to CAV16. Other viruses cultured in smaller numbers included CAV16, CAV4, CAV10, and CBV5, known etiologic agents of HFMD, as well as CAV6, CAV24, CBV3, CBV4, and EV18, cocirculating enteroviruses that may have caused at least some cases of HFMD. CAV6 was isolated from the vesicles of two patients and CAV24 from the vesicles of one patient. Some of these non-HEV71 enteroviruses could have played an indirect role in the HEV71 epidemic. Indeed, the possibility of HEV71 interacting with other enteroviruses in a previous HEV71 epidemic has been raised (*14*).

Among the patients with suspected enteroviral infection but without the classic symptoms of HFMD, the most frequently isolated virus was still HEV71. These cases represented the extremes of the clinical spectrum of HEV71, including nonspecific febrile illness in three patients and death from myocarditis and encephalitis in two patients. The other clinical presentations of the non-HFMD patients included aseptic meningitis, herpangina, and Guillain-Barré syndrome, conditions that could also be caused by HEV71. However, the viruses isolated from these patients were CBV4, CBV5, and herpes simplex virus 1. Of the total of 81 patients with culture evidence of HEV71 infection, most (93.8%) showed illness consistent with HFMD.

Until this epidemic occurred, no deaths had been associated with HFMD in Singapore, although HEV71-related deaths from encephalitis (*15*–*17*), pulmonary edema, and hemorrhage (*8*,*18*) have occurred elsewhere since the virus was first isolated in 1969 (*15*). In this epidemic in Singapore, the case-fatality rate among all reported HFMD case-patients was 0.08%, which is similar to the rate of 0.06% experienced in the 1998 Taiwanese outbreak (*9*).

Four deaths (two HFMD and two non-HFMD cases) were associated with HEV71. All occurred rapidly despite intensive care, within a day of the patient’s hospital admission, and after an average of 3.4 days of illness. The circumstances of these deaths were reminiscent of recent HEV71 deaths in the region (*10*,*18*–*20*). Of these four case-patients, three were autopsied, including a pair of siblings with HFMD and a patient with non-HFMD encephalitis. Their postmortem findings were similar, with HEV71 isolated from the brains of two case-patients and from the tonsils and intestines of the third. Whether HEV71 caused the death of the patient with myocarditis who was not autopsied is less clear since HEV71 was isolated from nonsterile sites (the throat and rectum), although the illness and epidemiology suggest the possibility.

During the epidemic, a fifth death occurred involving a boy with HFMD on whom an autopsy was conducted. No virus was cultured from him, possibly because of the advanced postmortem degradation of his tissues. However, HEV71 was likely also to have been the cause of death on the bases of the similarity of his clinical and postmortem findings to those of the siblings who died, as well as the epidemiologic links to age, time, and place.

Like other fatal HEV71 cases reported elsewhere (*7*,*16*,*20*,*21*), the primary pathologic changes found at autopsy of four case-patients in this study were in the brain, including the brainstem, which showed extensive inflammatory cell infiltrate and focal necrosis. In addition, pneumonitis was found in all the case-patients and myocarditis in two. In the Malaysian and Taiwanese outbreaks (*7*,*18*,*20*), however, no significant inflammation was found in the lungs of patients with fatal cases. Notably, the myocardium of 10 Malaysian patients was described as normal (*7*), whereas autopsy reports of 2 patients from the Taiwanese outbreak described mild myocarditis in 1 (*20*) and no myocarditis in the other (*18*).

HEV71 was cultured from the brain specimens of two of our autopsied case-patients, but viral cultures of the lung and heart were negative. Similarly, no HEV71 was isolated from 34 CSF samples studied, notwithstanding the diagnosis of aseptic meningitis. Besides encephalitis and death, other complications (such as aseptic meningitis and acute flaccid paralysis) have also been reported in other HFMD outbreaks (*7*,*9*). However, other than the three fatal cases and one case of aseptic meningitis, all HFMD cases in the Singapore epidemic were uncomplicated, despite the large number of patients.

Most HFMD patients were very young children (<4 years of age) with the peak incidence at 1 year, a finding consistent with other HFMD outbreaks (*7*,*9*,*22*,*23*). Male patients outnumbered female patients by 1.7 to 1. This predominance has been observed in other enteroviral infections in which the male-to-female ratio ranges from 1.5:1 to 2.5:1 (*24*). The reason for this finding is not clear but may suggest a susceptibility at the host genetic level. That two siblings died of HEV71 disease, which has a low case-fatality rate, further strengthens this suspicion. Further studies are warranted on the possible role of host genetic factors in the pathogenesis of HEV71 disease.

Since 1997, HEV71 outbreaks have occurred in Sarawak (*6*,*7*), the Malaysian Peninsula (*8*), Taiwan (*9*), Singapore (*25*), and Australia (*26*). To account for this wave of HEV71 outbreaks in the region, we suspected the presence of a susceptible population as a plausible explanation; however, HEV71 had appeared previously in Singapore in 1984 (*27*). The virus disappeared for a time, resurfacing initially in small numbers of patients in 1997–1999 (*25*); the number of infections then jumped in 2000. Because the same group of children with the highest incidence of infection in 2000 would have had been exposed to HEV71 since 1997, why a large outbreak did not occur earlier is unclear. Likewise, the large HEV71 outbreak in Taiwan (*9*) also took place when most of the population had apparent immunity. We suspect that changes in viral factors, including virulence and tropism, are possible factors in these occurrences.

The genetic sequences of the complete VP1 gene of HEV71 isolates from the four patients who died and three of the patients who did not, obtained during the Singapore outbreak in 2000, were compared in a recent study involving 66 HEV71 strains isolated between 1999 and 2001 from Malaysia, Singapore, and Western Australia (*28*). That study showed that the Singapore 2000 strains, like those isolated in 2000 in Sarawak, Malaysia, belong to genogroup B4, whereas the Singapore 1998 and Western Australia 1999 strains (from nonfatal case-patients) and Malaysian 1997 strains (including fatal case-patients) belong to the closely related B3 genogroup. The strains from the Taiwanese outbreak of 1998 were found to be in the more distantly related C2 genogroup. The viruses that caused fatalities in outbreaks in Malaysia (1997), Taiwan (1998), and Singapore (2000) were thus not genetically similar, at least in the VP1 region. These viruses did not belong to the same genogroup, which would have explained the similar characteristics of the outbreaks. Furthermore, although the same study suggests that a substitution of alanine with valine at position 170 of the VP1 region of genogroup C2 (lineage 1) strains may be associated with increased neurovirulence, no similar virulence-related mutation in the same genomic region was found for genogroups B3 and B4, to which the Malaysian and Singaporean fatal strains belong. No evidence exists from the deduced VP1 amino acid sequences of these two genogroups to link specific amino acid residues with the severity of illness or death. These observations indicate that the genetic determinants for virulence are still unclear.

Coinfection with a second virus has been suggested as yet another possible pathogenetic factor (*6*,*9*), and this theory is supported by the concomitant isolation of a subgenus B adenovirus with an enterovirus from three persons who died during a HFMD outbreak in Sarawak (*6*). Among the Singaporean patients with HEV71 infection, three had a second virus isolated concurrently. However, the presence of dual viruses did not result in severe disease, although a child with HEV71 and CAV16 coinfection died in Singapore in 1997 (*29*).

We considered whether any unusual medications, treatments, or dietary exposures contributed to the deaths. No evidence from the fatalities in Singapore suggests this possibility. Conversely, we reviewed whether any particular therapeutic modality improved clinical outcome, but found that this idea cannot be argued conclusively because the HFMD patient with aseptic meningitis recovered well with symptomatic treatment. The child who survived his two younger siblings had no complications from HFMD and was not cared for differently from his siblings before hospitalization. He was given prophylactic intravenous immunoglobulin solely because his siblings died. Whether this treatment, which was not administered to the patients who died, helped prevent severe disease in him is uncertain.

Considering that transmission of enteroviruses is mainly fecal-oral and through the respiratory route (to some extent) (*22*), we note that spread of the viruses is prevalent in child-care centers. To break the chain of transmission during the epidemic, the HFMD Task Force coordinated a swift, nationwide closure of preschool centers on October 1, 2000, reopening them on October 16, 2000, only when the HFMD reports recorded a declining trend, and no additional severe cases and deaths associated with the disease were reported. Other measures included repeated public health education through the mass media on observance of good personal hygiene, cleaning and disinfection of premises and articles both at home and at preschool centers, and keeping children away from crowds. These interventions may have played a role in bringing the epidemic under control by the end of October, although the outbreak may have also run its natural course by that time.

In September and October 2000, HEV71 caused the largest HFMD epidemic recorded to date in Singapore, an epidemic that involved mainly young children <4 years of age. Five deaths occurred, and HEV71 was isolated from four case-patients. Autopsies of four case-patients showed encephalitis, interstitial pneumonitis, and myocarditis. Virulence determinants of HEV71 and the precipitating factors for the epidemic itself unfortunately remain unknown. Based on our experiences during this epidemic, we found that an HFMD epidemic preparedness plan was useful in providing the framework for prompt actions to monitor the situation, identify the causative agent, interrupt virus transmission, and communicate with and solicit the cooperation of the media, parents, physicians, and preschool center personnel.
